# Isolation, characterization and identification of antibiofouling metabolite from mangrove derived *Streptomyces sampsonii* PM33

**DOI:** 10.1038/s41598-019-49478-2

**Published:** 2019-09-10

**Authors:** Venugopal Gopikrishnan, Manikkam Radhakrishnan, Thangavel Shanmugasundaram, Meganathan P. Ramakodi, Ramasamy Balagurunathan

**Affiliations:** 1 Centre for Drug Discovery and Development, Sathyabama Institute of Science and Technology (Deemed to be University), Jeppiaar Nagar, Chennai, 600 119 Tamil Nadu India; 2 Actinobacterial Research Laboratory, Department of Microbiology, Periyar University, Periyar Palkalai Nagar, Salem, 636 011 Tamil Nadu India; 3 DRDO-BU Centre for Life Sciences, Bharathiar University Campus, Coimbatore, 641 046 Tamil Nadu India; 4 CSIR-National Environmental Engineering Research Institute, Hyderabad Zonal Centre, CSIR-IICT Campus, Tarnaka, Hyderabad 500 007 India

**Keywords:** Environmental impact, Ocean sciences

## Abstract

In this study, we report the production, bioassay guided isolation and identification of antibiofouling metabolite from mangrove derived actinobacterium, *Streptomyces sampsonii* (PM33). The actinobacterial strain PM33 yields maximum amount of antifouling compounds through agar surface fermentation. In optimization, carbohydrates such as glucose, fructose and xylose, are suitable for maximum production of the active compound. In addition, other compounds such as malt extract, glutamine, and sodium chloride concentrations (2.5, 5 and 7.5%) and parameters such as pH 7.0 and temperature range 30 °C to 40 °C enhanced the production of antifouling metabolite. The antifouling metabolite was extracted in ethyl acetate. TLC and bioautography was used to separate and detect the antifouling metabolite present in the crude extract. The physico chemical features revealed that the antifouling metabolite PM33 – B as taxifolin (C_15_H_12_O_7_). The purified taxifolin was found to be active against biofouling bacteria, algal spore germination and mollusc foot adherence, respectively. Toxicity nature of taxifolin was also determined by adopting zebrafish embryos. The taxifolin isolated from mangrove-derived *Streptomyces sampsonii* PM33 is a promising candidate for the development of eco-friendly antifouling preparation.

## Introduction

Marine fouling is a common phenomenon to indicate the accumulation of marine life forms on wetted surfaces. Fouling, technically as well as economically, causes severe problems to shipping, offshore aquaculture and coastal industries around the world. To reduce the problems associated with fouling, metal-based or synthetic organic agents are used as antifouling coatings^1,2^. However, these antifoulants are found to be toxic and lethal to non-target lifeforms. In addition, the antifoulants could pollute the marine environment due to which the usage of these chemical based compounds has regulations. In these perspectives, development of novel eco-friendly antifouling compounds is timely needed^3^.

Research on antifouling metabolites from natural resources has been raised in recent time. Notably, marine organisms were found to be among promising resources for the production of non-toxic antifoulants. The special physical and chemical conditions of marine environment favour the diverse biological groups which possess a variety of structurally unique molecules with pharmacological activities^4^. Previous studies have reported several natural product antifoulants from marine organisms especially from invertebrates^5–7^. The studies carried out from 2009 to April 2014 reported 214 natural metabolites and 23 of synthetic derivatives with antifoulant activity. The structural analyses of these natural or synthetic compounds revealed that 82 compounds were novel^8^, which highlights the efficiency of marine organisms to produce antifouling metabolites. In addition, marine microbes have gained much attention and are being explored as potential sources for the production of environmental friendly antifouling metabolites. In comparison to marine invertebrates, marine microbes are the real sources for natural products including antifoulants^9^.

Actinobacteria are the group of Gram positive bacteria and have rich G + C composition in their DNA. They are the largest phylum among the bacterial domain which has the unique potential of producing novel metabolites for clinical and pharmaceutical applications^10^. The increasing numbers of literature on novel metabolites and the diversity of marine actinobacteria strongly support that they are the prolific source for novel high-value metabolites^11^.

Indian marine ecosystems are the promising source for bioactive actinobacteria with diverse biological activities^12,13^. Inspite of recent research on the marine actinobacterial antifouling metabolites showing the novel and precise mechanisms, the chemical structure of the compound, field assays and toxicity are yet to be defined^14,15^. In our earlier study, a quercetin molecule isolated from estuarine derived *Streptomyces fradiae* PE7 showed promising antifouling activity^16^. This study focuses on the antifouling potency of taxifolin, isolated from marine actinobacterial strain *Streptomyces sampsonii* PM33. Perhaps, this is the first study aiming to investigate the antifouling activity of taxifolin from marine actinobacteria.

## Results

### Characterization and taxonomy

**Figure 1:**
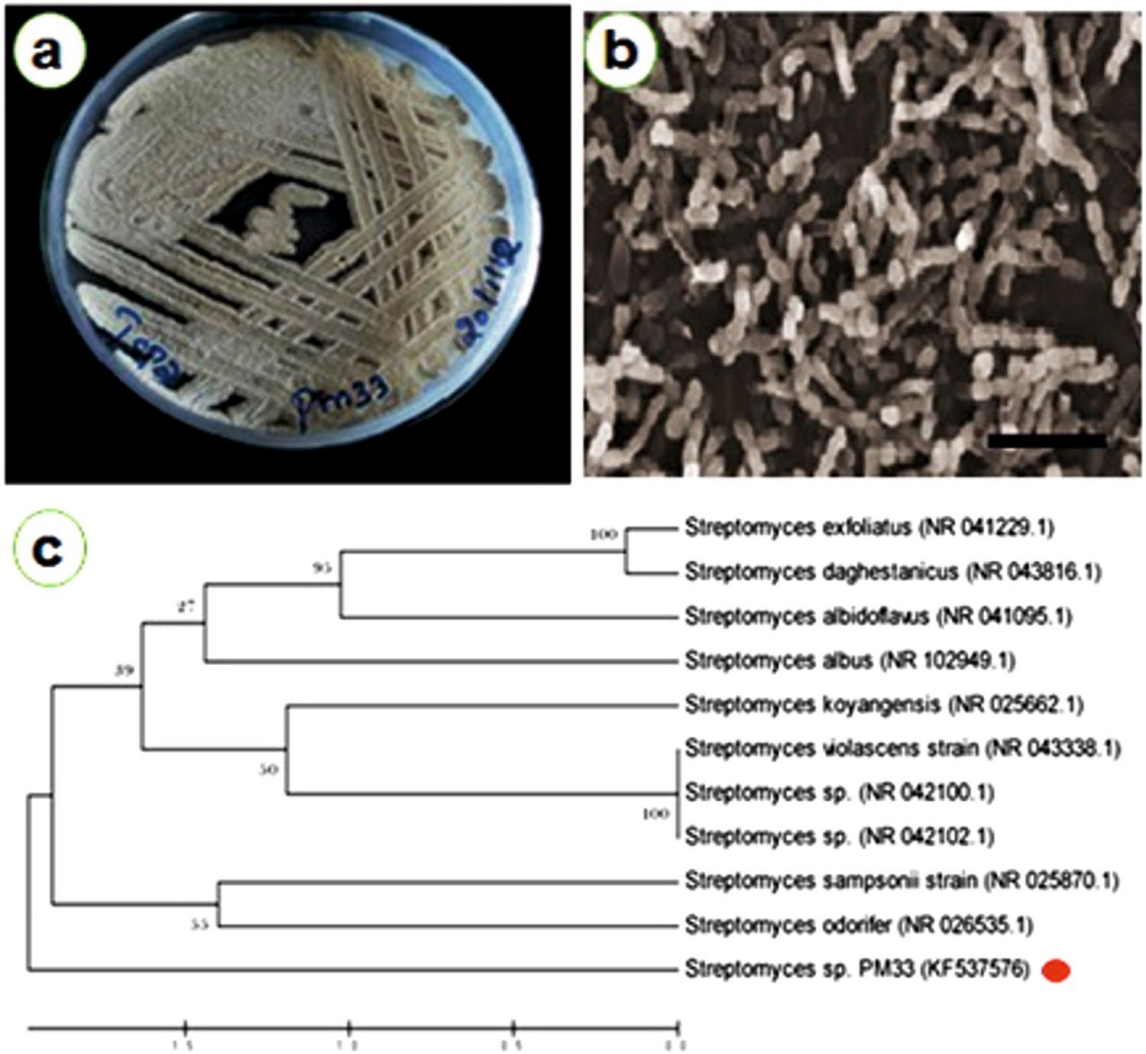
(**a**) Potential strain PM33, (**b**) Image of PM33 as obtained from Scanning Electron Microscope (1 µm) (**c**) Results of phylogenetic analyses showing the sister relationship between strain PM33 and *Streptomyces* sp.

Strain PM 33 showed both the non fragmented substrate mycelium and aerial mycelium with recti flexibile (RF) arrangement. Under scanning electron microscope, the spores appear with a smooth surface and oval shape (Fig. 1a,b). Cultural morphology on ISP2 agar was white leathery brown and showed brown pigment. Strain PM33 was also able to grow well on ISP4, ISP6 and ISP7 culture media. In addition, the strain PM33 showed moderate growth on ISP1, ISP3 and ISP5 media. This strain utilized a variety of carbon sources such as D-Glucose, D-Arabinose, D-Sucrose, D-Xylose, D-Inositol, D-Fructose, and D-Mannitol. Also good growth was observed at pH 7–11, temperature range from 20 °C to 40 °C and 2 to 7.5% NaCl. The enzyme assay showed that stain PM33 possess lipase, protease and amylase activities. The hydrolysates of PM33 had rich LL-diaminopimelic acid and glycine residues. The analyses of cell wall revealed no diagnostic sugars. The results suggested that the stain PM33 could be a *Streptomyces* species which was later confirmed by DNA sequence analyses. The 16S rRNA amplification of strain PM33 resulted in ~1311 bp sequence which was deposited in GenBank (KF537576). The BLAST analysis of 16S rRNA gene fragment revealed 99% similarity of PM33 strain to *S. sampsonii* ATCC 25495. The phylogenetic analyses (Fig. 1c) also showed that the strain PM33 is a member of *Streptomyces* genus.

### Production and extraction of antifouling metabolites

The maximum quantity of antifouling metabolites was produced through agar surface fermentation by the potential strain PM33. In the present study, various solvents were used to extract the antifouling compound and ethyl acetate was found to be suitable for the maximum extraction of crude compound from YEME agar. Also, the crude extract obtained using ethyl acetate exhibited maximum antifouling activity when tested against *Staphylococcus* sp-M1 and *Alcaligenes* sp. The results of the activity of extract obtained from different solvents are shown in Table 1. The solvent n-hexane did not yield any extract from the culture medium.

**Table 1:** Efficiency of different solvents for extracting antifouling compound from the actinobacterial strain PM33.

Solvents	YEME broth			YEME agar		
	Quantity*	*Staphylococcus* sp-M1	*Alcaligenes *sp.	Quantity*	*Staphylococcus* sp*-*M1	*Alcaligenes *sp.
Chloroform extract	10	11 ± 1	12 ± 2	21	12.33 ± 1.52	12 ± 1.73
Ethyl acetate extract	23	16 ± 1	17.66 ± 1.52	37	21 ± 1	18 ± 1.73
n-Hexane extract	—	0 ± 0	0 ± 0	—	0 ± 0	0 ± 0
Methanol extract	—	ND	ND	35	13.66 ± 1.52	12.33 ± 2.08

ND – Not Determined; *[mg/100 ml].

### Medium optimization

This study also tried to optimize the parameters required for the production of maximum quantity of antifouling metabolites from strain PM33 by adjusting various factors. Production of antifoulants from strain PM33 by using various factors is given in Table 2.

**Table 2:** Influence of fermentation parameters on the production of antifoulant by actinobacterial strain PM 33.

Parameters	Variables	Growth	Quantity of crude extract (mg/100 ml)	Activity* against *Staphylococcus* sp-M1
Carbon source	D-Glucose	Good	28	15
	D-Arabinose	Poor	9	7
	D-Sucrose	Poor	8	7
	D-Xylose	Poor	9	8
	D-Inositol	Moderate	15	11
	D-Mannitol	Moderate	14	12
	D-Fructose	Good	23	15
	D-Rhamnose	Moderate	19	11
	D-Raffinose	Moderate	17	11
	D-Cellulose	Moderate	13	10
Nitrogen source	D-Asparagine	Moderate	15	12
	D-Glutamine	Good	26	17
	D-Tyrosine	Poor	9	11
	Malt extract	Good	32	16
	Peptone	Moderate	21	10
	Beef extract	Moderate	17	13
pH	5	No growth	—	—
	7	Good	29	16
	9	Good	28	12
	11	Moderate	21	12
Temperature	20	No growth	—	—
	30	Good	33	15
	40	Moderate	32	15
	50	—	—	—
Minerals	MgSO_4_	Poor	8	0
	KNO_3_	Moderate	14	7
	KCl	Moderate	13	8
	MgCl_2_	Moderate	13	7
	FeSO_4_	Poor	7	—
NaCl %	0	Poor	7	—
	1	Moderate	11	—
	2.5	Good	29	16
	5	Good	30	15
	7.5	Poor	12	9
	10	No Growth	—	—

### Isolation and characterization of antifouling metabolite

The antifoulant activity of the purified metabolite was studied using TLC-agar overlay bioautography method. The results of bioautography illustrated the ability of separated metabolites (spots) to inhibit the growth of biofouling bacteria. In analytical TLC, the extract yielded four spots with the solvent system n-hexane: ethyl acetate (2:3). The R_*f*_ measure of four separated metabolites was estimated to be (A) 0.91, (B) 0.78, (C) 0.34 and (D) 0.25, respectively. In bioautography, the metabolite that was separated in the second place (PM33-B) inhibited the growth of a biofouling causing organism *Staphylococcus* sp*-*M1. The metabolites were separated from TLC and 125 mg of active metabolite PM33-B was obtained from 1 g of crude extract (Supplementary Fig. S1).

### Physico-chemical properties and structure of active metabolite

**Figure 2:**
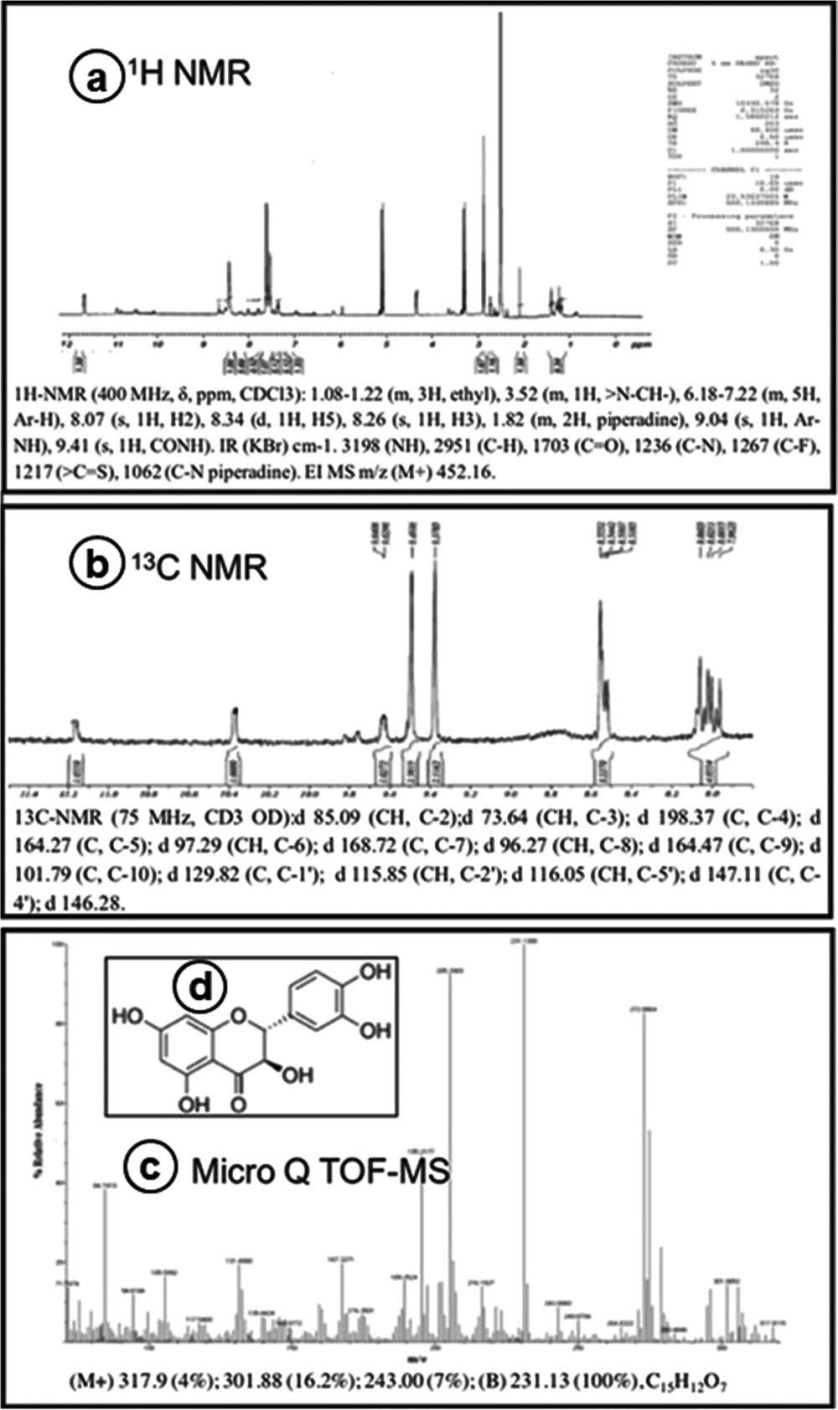
(**a**) Spectrum obtained from ^1^H Nuclear Magnetic Resonance spectroscopy, (**b**) Results of ^13^C NMR analyses for compound PM33-B, (**c**) LC-MS results for compound PM33-B, (**d**) Structure of Taxifolin compound purified from strain PM33.

The active metabolite PM33-B was found to be soluble in solvents such as ethyl alcohol, methanol, water, Dimethyl sulfoxide, Dimethyl formamide, and ethyl acetate but the metabolite was insoluble in n-hexane. The chemical analyses of the active metabolite illustrated that PM33-B to be a flavonoid metabolite. The metabolite had strong absorption spectra at 400 nm when analyzed in UV-visible spectrophotometer. The FT-IR analysis showed that the PM33-B have broad absorption at 3380 cm^−1^ and 3088 cm^−1^ which denotes the presence of a OH-group. The absorption peak at 1100 cm^−1^ indicates the CH-stretching vibration of the aromatic group. The peak at 3088 cm^−1^ illustrates the C-H bending for CH group. The FT-IR spectrum is showed in Supplementary Fig. S2. The ^1^H-NMR analysis of PM33-B (Fig. 2a) showed a doublet at d 8.34 ppm and two singlets at d 3.56 ppm and d 3.52 ppm, respectively. The doublet indicates the presence of -HC=CH- group while the singlets at d 3.56 ppm and d 3.52 ppm reveal-HC=CH- and CO-CH groups, respectively. The ^13^C-NMR analysis of PM33-B illuminated signal at 187.42 ppm which illustrate the carbonyl carbon in PM33-B (Fig. 2b). The molecular weight of PM33-B was measured to be 304.25 (Fig. 2c). Mass spectrum has showed m/e (% relative abundances): (M+) 317.9 (4%); 301.88 (16.2%); 243.00 (7%); (B) 231.13 (100%). Based on the physico-chemical properties the compound PM33-B was identified as (2R, 3R)-2-(3,4-dihydroxyphenyl)-3,5,7-trihydroxy-2,3-dihydrochromen-4-one with the molecular formula of C_15_H_12_O_7._ The molecular structure of PM33-B is shown in Fig. 2d. The physico chemical properties and the literature survey revealed the compound PM33-B as Taxifolin.

### Antifouling assay

**Figure 3:**
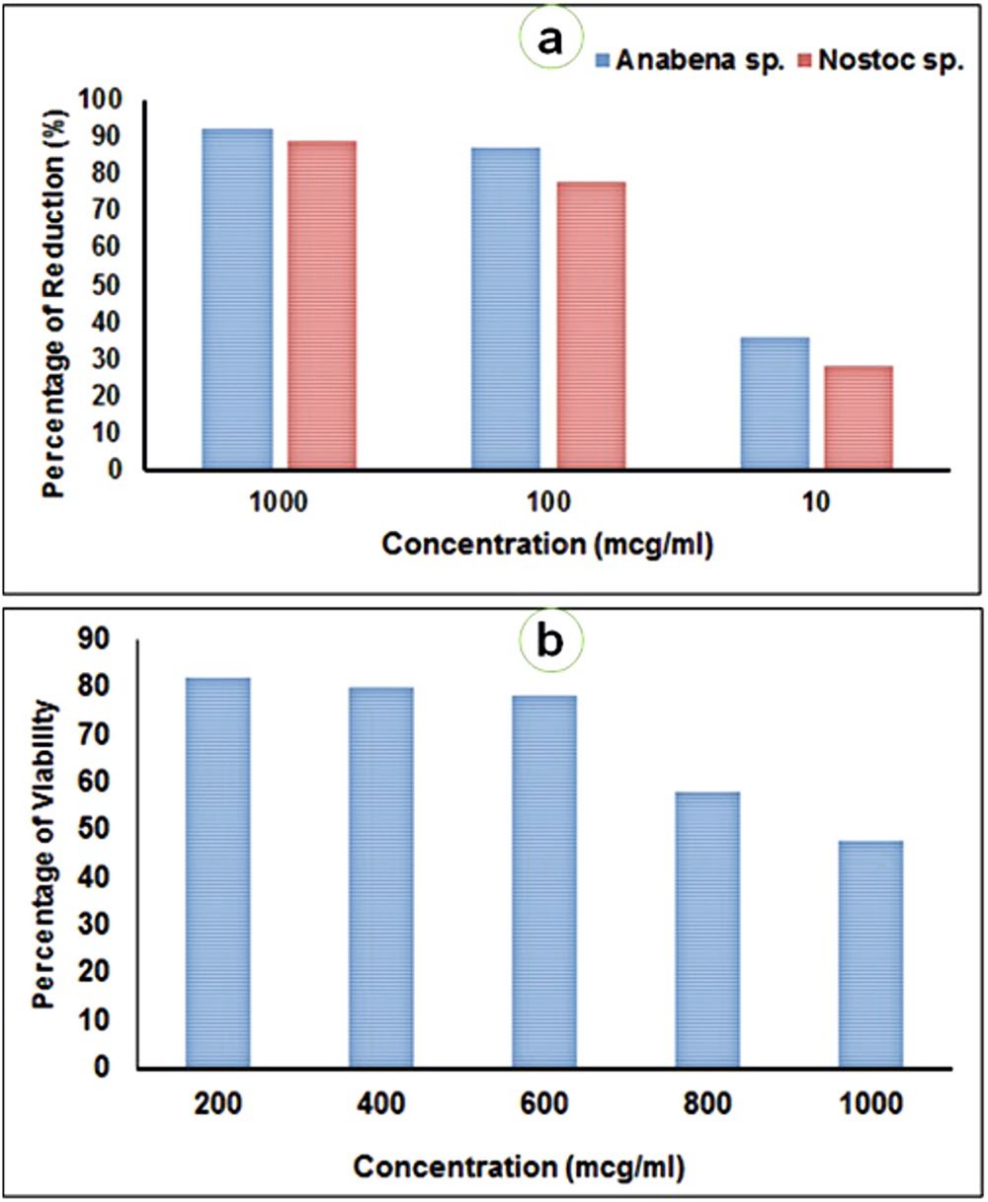
(**a**) Results showing anti-algal germination activity by PM33-B, (**b**) Results showing activity of taxifolin isolated from PM33 against foot adherence of *Perna indica*.

**Figure 4:**
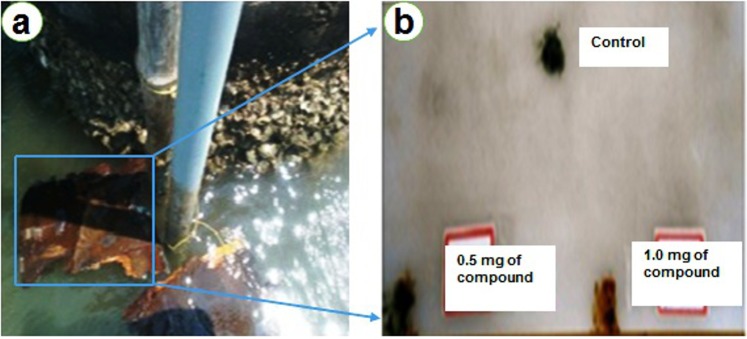
Field experiment results of the anti-biofouling compound, Taxifolin on (**a**) marine surface and (**b**) PVC panels with control.

The purified taxifolin showed significant antifouling activity against biofouling bacteria with the MIC values ranging from 1.6 to 25 µg/ml (Supplementary Table 1). PM33-B exhibited potential antifouling activity when tested against *Anabaena* and *Nostoc* sp. In addition, taxifolin (100 µg/ml) reduced >70% of algal spore germination (Fig. 3a**)**. In mollusc foot adherence assay, PM33-B showed its maximum antifouling activity after 24 h and 48 h. In addition, some molluscs were dead in the test group which could be due to toxic effect of compound. The control group showed normal attachment of mollusks (Fig. 3b). In field assay, the taxifolin compound coated test PVC panel did not have the adherence of microfouling or macrofouling organisms. Nonetheless, the test wooden boat showed lesser adherence or settlement of biofouling organism after two weeks in comparison to controls. The PVC panels with taxifolin compounds of PM33 had smaller cover of biofouling organisms even after 4 weeks whereas the wooden surface coated with taxifolin compound has showed less biofouling formation organisms after two weeks (Fig. 4).

### Toxicity testing in zebrafish model

**Figure 5:**
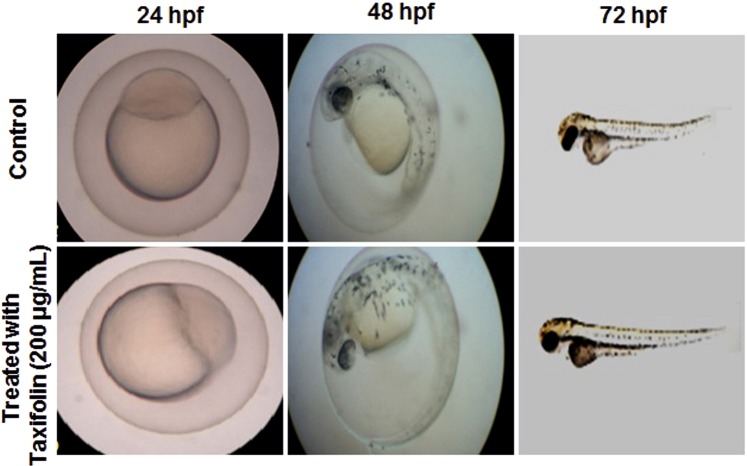
Toxicity study of anti-biofouling compound, Taxifolin on Zebrafish model up to 72 h.

The toxicity assay was carried out using zebra fish model. The study showed that the zebrafish embryos treated with taxifolin had pericardial edema around the heart. Also, after incubation with taxifolin, the zebrafish embryos had poor blood circulation in addition to small brains and small eyes. An increase in the severity of these symptoms was observed when the amount of taxifolin was increased. Taxifolin with 0.5 µg/ml and 1 µg/ml concentrations caused pericardial edema in 0.6% of zebrafish embryos. But when the concentration of taxifolin was increased to1.5 µg/ml and 2.0 µg/ml, 60% (12 out of 20) of zebrafish embryos had pericardial edema. The results suggest that taxifolin possess less toxic effects in lower concentrations (Fig. 5).

### Testing for *in vitro* cytotoxicity on normal lung cell line

**Figure 6:**
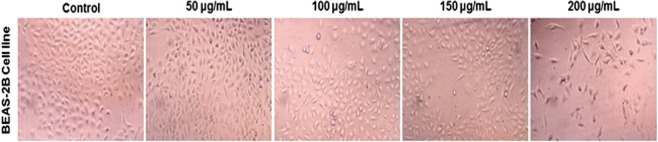
Toxicity study on BEAS – 2B Cell line at different concentration.

The results of toxicity effect on Zebrafish model showed that the taxifolin has less toxic effect at lower concentrations. Taxifolin exhibited less inhibition potential in Bronchial Epithelial cell line BEAS – 2B cell lines. Taxifolin showed the LC_50_ range between 150 µg/mL and 200 µg/mL in the BEAS – 2B cell lines (Fig. 6).

## Discussion

From a natural products perspective, marine microbes are better resources for novel antifoulants to control biofouling in marine structures. However, studies aiming to explore antifoulants from marine actinobacteria are sparse. Recently, few publications described the potential of marine *Streptomyces* to produce antifouling metabolites that is active against micro and macrofouling organisms^16,17^. Nonetheless, continuous exploration of marine organisms for novel antifoulants is worthy due to the growing technological or economical concerns associated with fouling organisms. In this view, this study attempted for the isolation, characterization and identification of potential antifouling metabolites effective against marine biofouling organisms. Specifically, this study reports the isolation, characterization and potential antifouling activity of taxifolin from *Streptomyces* sp. PM33 isolated from mangrove sediments. A combination of physiological parameters, chemotaxonomic characteristics, 16S rRNA gene sequences and phylogenetic analysis indicated that the closest relatives of study strain PM33 is *Streptomyces sampsonii* ATCC 25495. It is noteworthy that many studies reported many flavonoid compounds from actinobacteria including *Streptomyces*, but this is the first report to show taxifolin from actinobacterial sources.

Large amount of pharmaceutically valuable antibiotics were produced from *Streptomycetes* via submerged fermentation in bioreactors^18,19^, but the compounds required subsequent purification and identification process. One of our previous studies on *Streptomyces fradiae* PE7 reported that agar surface fermentation using YEME media was efficient for producing antifouling compound as compared to submerged fermentation on YEME broth^20^. In this study agar surface fermentation was adopted method for the production of antifouling metabolites and showed promising metabolite production.

In the present study, the conditions were optimized by using ASF which identified economically effective components for producing antifoulants. Specifically, classical one factor at a time method was adopted to identify the interaction of the important factors for enhancing the production of the antifouling compound as previous researches reported that the optimization technique maximizes the antifouling compound production^16,17,21^.

*Streptomyces sampsonii* and its bioactivity is less reported^22–24^. In addition, the antifouling activity of *Streptomyces sampsonii* is understudied. In this context, this study analyzed the antifouling compound from PM33 which is closely related to *S. sampsonii*, hence, demonstrates the ability of *S. sampsonii* to produce antifouling compounds.

The chemical analyses showed that the compound isolated from PM33-B is a flavonoid. Specifically, the compound PM33-B was found to be taxifolin. An antifouling agent isolated from natural sources could act as an ideal antifoulant in many ways on the target organisms. In general, the antifouling agent should exhibit the potential to fight against biofilm formation. Specifically, Inhibition of algal spore germination and mollusc foot adherences are the main mechanism of antibiofilm activities of the compound. In this study, the taxifolin compound isolated from PM33 inhibited algal spore germination. Also, the taxifolin showed effectiveness against also mollusc foot adherences. Importantly, the field experiments revealed good antifouling activity of taxifolin, when tested on wooden surface and PVC panels. These analyses showed the antifouling potential of taxifolin isolated from strain PM33. Further toxicity assay based on Zebra fish models revealed the less or moderate toxicity of taxifolin compound isolated from PM33. Natural flavonoids are characterized by their antioxidant, pharmacological, anti-inflammatory, anti-allergic, antiviral, anticarcinogenic, as well as therapeutic and cytotoxic properties which possess a wide range of industrial applications^25^. Thus, the taxifolin isolated from mangrove *Streptomyces sampsonii* PM33 has a great commercial feature in the future.

## Materials and Methods

### Actinobacterial strain

The actinobacterium PM33 was isolated from the sediment collected from Vellar estuarine (Lat 11°29′N; Long 79°46′E), Parangipettai, Tamil Nadu, India. The methods of sample collection and location coordinates are discussed in^16^. During the preliminary screening, strain PM33 was exhibited significant activity against (*Bacillus* sp., *Aeromonas* sp., *Micrococcus* sp*, Alcaligenes* sp*., Lactobacillus* sp. *Staphylococcus* sp. *Pseudomonas sp*. and *Vibrio* sp.) marine biofouling bacteria^20^.

### Characterization and taxonomy

Microscopic, cultural and physiological and cell wall characteristics of strain PM33 was studied following standard method^26–30^. The 16S rRNA gene sequence was used to study the molecular characterization of the potent strain PM33. The 16S rRNA gene sequence of PM33 was sequenced and the sequence was submitted to GenBank (KF537576).

### Production and extraction of antifouling metabolites

Production of antifouling metabolites from strain PM33 was conducted by both agar surface and submerged fermentation methods. For surface fermentation method YEME agar was used whereas YEME broth was used for submerged fermentation. The fermentation reactions were carried out at 28 °C for 5 days and the crude extract of strain PM33 was recovered from the agar medium as well as from the cell-free supernatant by using different organic solvents. The crude extract in solvents was concentrated using evaporation technique and the residue thus obtained was used to determine antifouling activity against fouling bacteria following standard disc diffusion method. In this study, the bacterium *Staphylococcus* sp-M1, isolated from marine fouling samples^16,20^ was used to test the antifouling effect of crude extract.

### Medium optimization

A classical one factor variable at a time, with remaining parameters kept unchanged, was followed to study the effect of chemical composition of media and cultural states on the production of antifouling compound by using one factor at a time^31^. Supplementation tests were carried out to analyze the enhancement or inhibition of antifouling compound due to nutrients. In supplementation experiments sources like carbon, nitrogen, and amino acids were supplemented in the basal medium which consists of 1% dextrose, 1% yeast extract and 0.5% NaCl with the pH 7.0. The potential effect of the nutrient on the production of antifouling compounds was studied. The crude extract obtained from the media was tested against marine fouling bacterium *Staphylococcus* sp-M1.

### Isolation of antifouling compounds from crude extract

Maximum quantity of crude extract from PM33 was obtained from YEME agar with ethyl acetate^32^. The active metabolite present in the crude extract was purified using analytical thin layer chromatography on silica gel coated plates using solvents with different polarity in different ratio. The active metabolite separated in TLC was identified by direct bioautography using *Staphylococcus* sp-M1 as test organism^17,33^.

### Physico-chemical characterization and structure elucidation

The biochemical assays as mentioned in^34^ was followed to determine the chemical class of purified metabolite. The solubility of pure metabolite was analyzed using various solvents such as distilled water, ethyl acetate, chloroform, methanol, acetone, petroleum ether, dichloromethane and diethyl ether at 1 mg/ml concentration. The chemical groups/characteristics of antifouling metabolite including aliphatic, aromatic, saturated nature, unsaturated nature, presence of elements and the functional groups of compounds were studied using UV-Vis spectrophotometer at 190–900 nm (Shimadzu UV-1700), FT-IR spectrum was obtained between 4,000 and 450 cm^−1^ (Shimadzu 8300) and the metabolite was analyzed on ^1^H NMR and ^13^C NMR with Bruker DRX-500 (500 MHZ). The molecular weight of the metabolite was determined by analyzing the purified active metabolite on LC-MS (Shimadzu).

### Antifouling assays

#### Activity of purified metabolite on biofouling bacteria

The antifouling efficacy of purified metabolite was tested against twelve bacterial strains that were isolated from marine fouling samples in the earlier study^20^, using disc diffusion assay (100 µg /disc) following the method described in^35^.

#### Activity of purified compound against cyanobacterial spore germination

The suspension of macro spores of biofouling causing algae *Anabaena* sp. and *Nostoc* sp. were used to determine the inhibitory effect of the purified compound on algal spore germination. Briefly, the purified metabolite at different concentrations (1000 μg/ml, 100 μg/ml and 10 μg/ml) were mixed with algal spores in microtitre plate, separately and the plates were kept in dark condition for 6 h. Later, the plates were incubated at 18 °C for 16 h under light followed by 8 h incubation in dark condition. The light: dark incubation cycle was continued for six days. After six days of incubation, a drop of spore suspension treated with active compound was observed under light microscope (400×) to estimate germinated spores and the percent reduction of germination as described earlier^36^.

#### Activity of purified compound against Mollusc foot adherence

The activity of purified compound against Mollusc foot adherence was studied by using the samples of *Perna indica*. Primarily the stock of *P. indica* were collected from Parangipettai coastal area, India and acclimatized in the laboratory for 24 h. From the collected molluscs five healthy *P. indica* were selected and placed on a polyvinyl Petri plate and added 20 ml of seawater with various concentrations (200–1000 μg/ml) of taxifolin compound. Five healthy *P. indica* were maintained in seawater without extracted compound as controls. The experiments were carried out in triplicate with mild aeration. The tests and controls were not supplemented with any additional feed during the assay period. After 24 hours, the anti-adherence property of extract on molluscs was studied following the method described^37^.

### Field assay

The field evaluation tests were also carried out using the wooden parts of boats following the modified method of ^38^. Briefly, 1.52 g phytogel (Sigma-Aldrich Inc., USA) with 35 ml of distilled water and 0.5 ml the purified antifoulant of strain PM33 was mixed. Later the gel along with antifouling compound was spotted on PVC panels. Also, the hardened gel was applied as substratum on the wooden boat. Finally, the PVC panels and the wooden parts were kept in the jetty area (Parangipettai, Near Chidambaram, Tamil Nadu, India). The details of preparation of phytogel with antifouling compound are described in^16^. The activity of antifoulant was estimated once in every 15 days as described earlier^39^.

### Toxicity testing in zebrafish model

Post embryo eggs of Zebrafish were taken and kept in E3 medium with 1% methylene blue. Different concentrations of purified compound (0.5, 1.0, 1.5 and 2.0 µg/ml) were prepared and inoculated in 20 ml of F3 medium containing 20 eggs. Zebrafish embryos were observed frequently under light microscope as described in^40^ and^41^ to understand the effect of purified compound on phenotype and development of the embryos. We are confirming that all experiments were performed in accordance with relevant guidelines and regulations of Periyar University Institutional Animal Ethical Committee (IAEC, 1085/PO/Re/S/07/CPCSEA).

### Testing for *in vitro* cytotoxicity on normal lung cell line

Cytotoxic activity of the purified compounds was tested by MTT assay on BEAS – 2B cell line, obtained from National Centre for Cell Sciences (NCCS), India, at 100 µg, 10 µg, 1 µg, 0.1 µg and 0.01 µg concentrations as recommended by^42^. The test was carried out at Sri Ramachandra University, Chennai.

## Conclusions

This is the first report to show the ability of *Streptomyces* sp. to produce taxifolin. Our present research has shown that taxifolin displays various antifouling effects as the taxifolin also inhibited the algal spores and attachment of molluscs. These findings strengthen the applications of taxifolin isolated from marine-derived *Streptomyces sampsonii* (PM33) as antifouling agent. Taxifolin could be an eco-friendly antifouling agent to prevent the adherence of diverse fouling organisms on marine surfaces. However, additional field studies need to be designed in the future to check the effectiveness of taxifolin on marine environment to support the taxifolin from *Streptomyces* as a replacement of presently available chemical antifoulants.

## Supplementary information

Supplementary files
