# Supplementary material for: Isolation, characterization and identification of antibiofouling metabolite from mangrove derived Streptomyces sampsonii PM33
Source: Sci Rep. 2019 Sep 10;9:12975. doi: 10.1038/s41598-019-49478-2 (PMC6736841; doi:10.1038/s41598-019-49478-2)
Supplement: Supplementary file 1 — Supplementary files [file 41598_2019_49478_MOESM1_ESM.docx]

**Supporting information**

**Isolation, characterization and identification of antibiofouling metabolite from mangrove derived *Streptomyces sampsonii* PM33**

VenugopalGopikrishnan^a,b^, ManikkamRadhakrishnan^a^_,_ThangavelShanmugasundaram^c^,Meganathan P. Ramakodi and RamasamyBalagurunathan^b,^*

**Supplementary Table 1: Minimum Inhibitory Concentration of PM33-B against different biofouling bacteria**

**
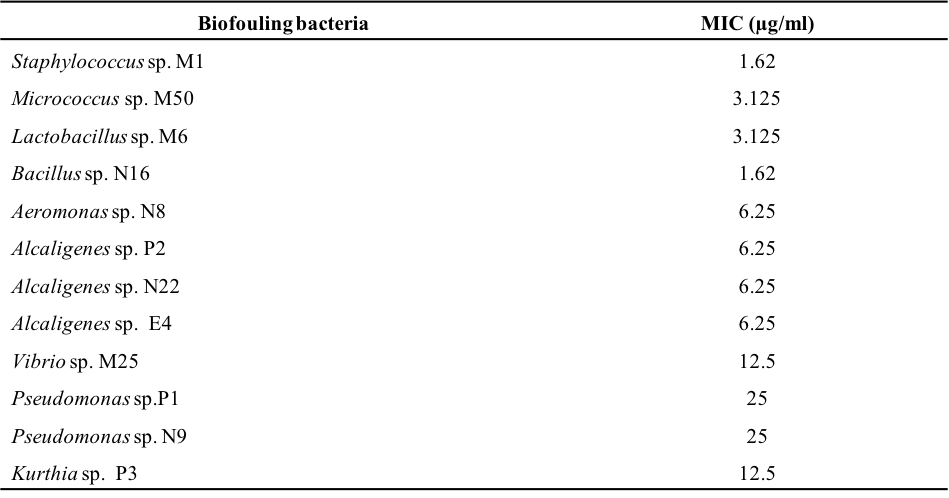
**

**Supplementary Fig. S1.** Purification and bioautographicresults of compound PM33-Bbased on Thin Layer Chromatography


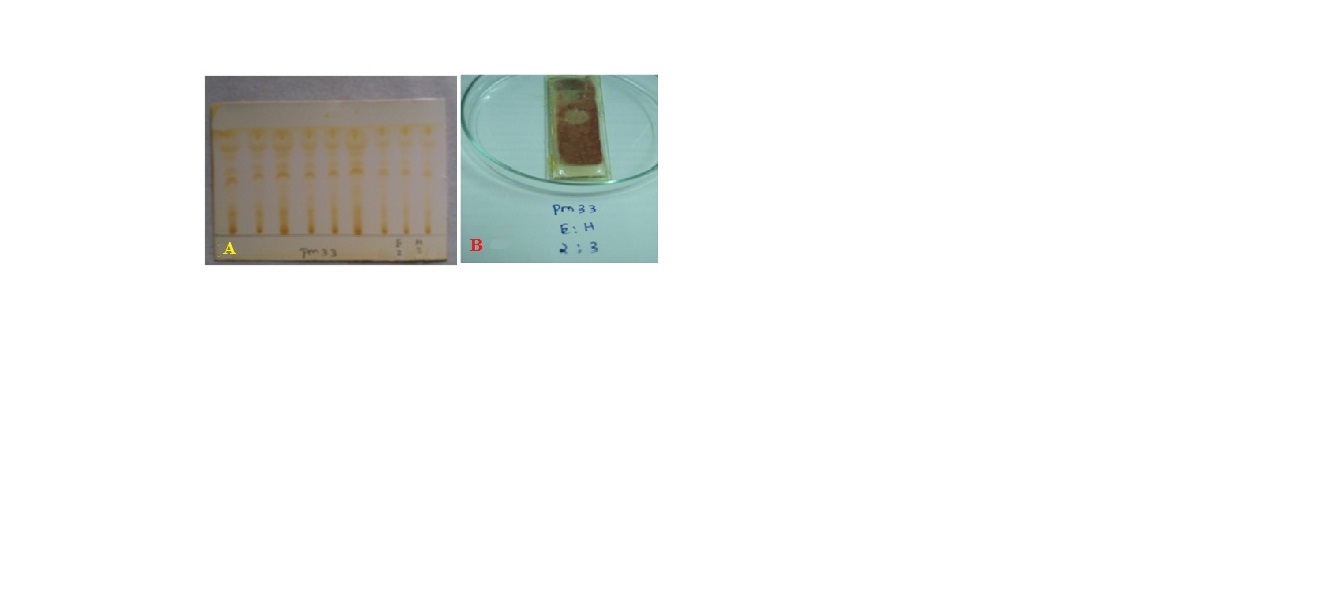


PM33-B

PM33-B

**Supplementary Fig. S2.** UV absorption and FT-IR spectrum of Taxifolin compound

**
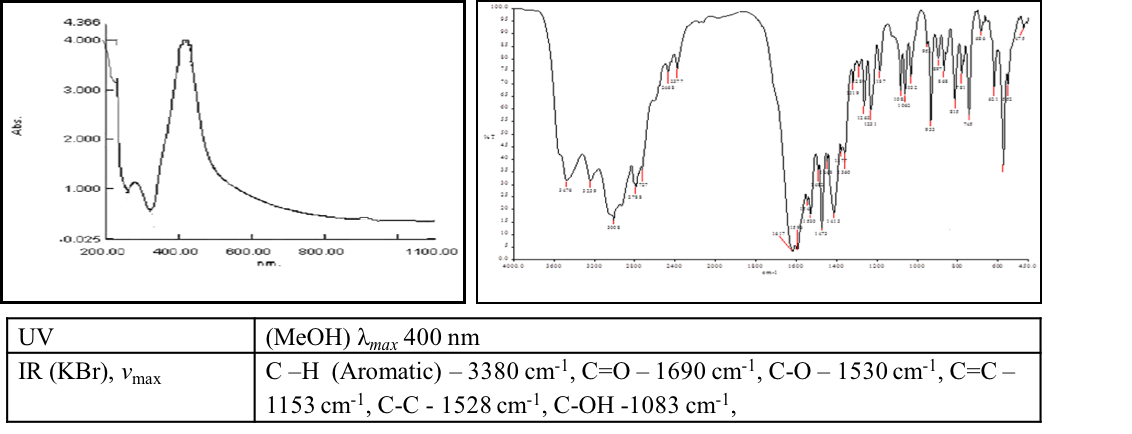
**
